# Large cross-effect dynamic nuclear polarisation enhancements with kilowatt inverting chirped pulses at 94 GHz

**DOI:** 10.1038/s42004-023-00963-w

**Published:** 2023-08-22

**Authors:** Yujie Zhao, Hassane El Mkami, Robert I. Hunter, Gilles Casano, Olivier Ouari, Graham M. Smith

**Affiliations:** 1 School of Physics and Astronomy, University of St Andrews, KY16 9SS Fife, Scotland; 2 Aix Marseille University, CNRS, ICR, UMR 7273, F-13013 Marseille, France

**Keywords:** Solution-state NMR, Physical chemistry, Solution-state NMR, Structure elucidation

## Abstract

Dynamic nuclear polarisation (DNP) is a process that transfers electron spin polarisation to nuclei by applying resonant microwave radiation, and has been widely used to improve the sensitivity of nuclear magnetic resonance (NMR). Here we demonstrate new levels of performance for static cross-effect proton DNP using high peak power chirped inversion pulses at 94 GHz to create a strong polarisation gradient across the inhomogeneously broadened line of the mono-radical 4-amino TEMPO. Enhancements of up to 340 are achieved at an average power of a few hundred mW, with fast build-up times (3 s). Experiments are performed using a home-built wideband kW pulsed electron paramagnetic resonance (EPR) spectrometer operating at 94 GHz, integrated with an NMR detection system. Simultaneous DNP and EPR characterisation of other mono-radicals and biradicals, as a function of temperature, leads to additional insights into limiting relaxation mechanisms and give further motivation for the development of wideband pulsed amplifiers for DNP at higher frequencies.

## Abstract (web summary)

Dynamic nuclear polarization is a technique to enhance NMR signals, often achieved by continuous-wave irradiation of the samples. Here, the authors create large polarization gradients by using high-power chirped inversion pulses at 94 GHz at an average power of a few hundred mW with fast build-up times, reaching NMR signal enhancements of up to 340.

## Introduction

Dynamic nuclear polarisation (DNP) is a technique used to enhance NMR signals by transferring magnetic polarisation from electrons to target nuclei. This enhancement can be large and has a theoretical maximum dictated by the ratio of the electron and nuclear gyromagnetic ratios. There are a number of distinct DNP mechanisms that have been successfully applied including: the Overhauser effect; the solid-effect; thermal mixing; and the cross-effect^1,2^. The Overhauser effect relies on fast dynamics and cross-relaxation between coupled spins and is mostly applicable to metals or liquids^3–6^. The solid-effect is a two-spin process and is based on exciting forbidden transitions and typically requires mono-radical polarising agents with narrow lines, such as trityls^7^, where allowed and forbidden transitions do not overlap. Thermal mixing^8–11^ needs strongly coupled spin networks, and requires electron dipolar frequencies that are comparable to the nuclear Larmor frequency. However, it is the cross-effect that has arguably had the most impact on magic angle spinning (MAS) solid-state NMR, usually implemented in the 80–120 K temperature range^12,13^. In this method, microwaves are used to create a polarisation gradient across an inhomogeneously broadened line by exciting allowed electron transitions; a process which does not typically require high average power and is often optimised for MAS with biradical polarising agents.

Cross-effect DNP was first discovered by Kessenikh^14,15^ in 1963, and later independently found by Hwang and Hill^16,17^ in 1966. However it was the seminal work of the Griffin group at MIT which laid the foundations of modern high-field DNP, by making use of continuous wave (cw) gyrotrons as microwave sources that were able to provide a few watts of power at hundreds of GHz. This group established the viability of the technique for solid-state NMR, initially demonstrating enhancements of ~ 50 at 100 K with 4-amino TEMPO and establishing the importance of the glassy properties of “DNP juice” to maximise enhancements^18–20^. In collaboration with other groups they then demonstrated the importance of biradicals like TOTAPOL for MAS^21,22^. Later, the development of more sophisticated biradicals, for high-field MAS^23–25^, established new DNP enhancement records^26–29^.

The cross-effect is based on allowed flip-flop-flip transitions involving two coupled electrons at resonant frequencies *ω*_1*e*_ *and ω*_2*e*_ and a local nuclear spin at frequency ${\omega }_{n}$, where the frequency difference between the electrons corresponds to the nuclear Zeeman frequency $(\left|{\omega }_{1e}-{\omega }_{2e}\right|={\omega }_{n})$. It is applicable to electron spin systems with inhomogeneously broadened lines, such as nitroxides or transition metals, which are broad compared to the nuclear Zeeman splitting. At high electron concentration the probability of such flip-flop-flip transitions can be surprisingly high^7^, and is a dynamic process that occurs naturally in the absence of resonant microwave radiation. Under thermal equilibrium conditions, flip-flop-flip and flop-flip-flop transitions between any suitable pair of coupled electrons and nuclei are equally likely, and there is no net bias to preferentially drive nuclei to flip in one direction, and therefore no net DNP effect is observed. However, the necessary bias can be achieved by saturating (or inverting) part of an inhomogeneously broadened line, to create an electron polarisation gradient across the line. The local NMR enhancements can then be positive or negative depending on the direction of the gradient, and nuclear polarisation can further spread via nuclear spin diffusion. Saturation of part of the line, to create the polarisation gradient, is conventionally achieved with cw or continuous frequency swept radiation, often in conjunction with MAS.

There is now a far better understanding of some of the subtle trade-offs involved in cross-effect DNP experiments^30–37^, and a large number of different polarising agents have been synthesized and evaluated, with a view to optimise: biradical concentration^38^, electron relaxation times^26^, solvent accessability^39^ and the balancing of exchange and dipolar coupling in biradicals^29,40^.

More recently, a number of groups have also explored the benefits of chirped or low-power pulsed radiation^41–44^ to more precisely saturate parts of the spectrum to create the necessary polarisation gradient. This has generally been implemented by frequency modulating solid-state sources with fast arbitrary waveform generators (AWGs)^45,46^ at cw output power levels of no more than a few watts. These static experiments have often been performed at low temperatures (< 30 K) where it is easier to saturate the spins; where the benefits of swept frequency excitation have been clear^45–52^. They are also predicted to be highly beneficial under MAS conditions^53^.

A key idea in static cross effect DNP is that the maximum attainable nuclear polarisation is governed by the difference in electron polarisation between two sets of coupled spins that are separated in resonant frequency by the nuclear Larmor frequency. In the high-temperature approximation, microwave saturation of one set of spins is expected to give a maximum nuclear polarisation enhancement of 329. However, fully inverting the spins, in principle, increases the maximum possible enhancement to 658^54^. Recently it was proposed that even higher polarisation gradients, and hence DNP enhancements, could be achieved by optically exciting a chromophore to a hyperpolarised state within a chromophore/nitroxide spin pair^55^.

Although larger polarisation gradients can be expected to result in more efficient polarisation transfer, it should be noted that spin inversion is inherently a transient process. A large polarisation gradient is maintained only for a short period followed by a necessary recovery period back towards thermal equilibrium. It is therefore perhaps not immediately obvious why inversion pulses should represent an optimal excitation strategy, as the nuclear polarisation will decrease during the relatively long recovery period. However, the recovery time also has two additional important benefits. Firstly it allows time for nuclear spin diffusion, which does not require an electron polarisation gradient. Secondly it reduces the effect of spectral diffusion, which otherwise limits the achievable polarisation gradient.

In this work, we show that high-power chirped inversion pulses at comparable average powers to cw frequency modulation does lead to increased cross-effect DNP efficiency. Measurements are performed using a 94 GHz pulsed AWG EPR spectrometer, known as HiPER. This incorporates a high-power extended interaction klystron amplifier (EIKA), which provides more than 1 kW of pulsed power at 94 GHz with effective B_1_ fields of ~ 15 G (nutation frequencies of 42 MHz) at the sample. The integration of an AWG allows considerable flexibility in specifying phase and amplitude modulated pulses over a 1 GHz instantaneous bandwidth. The spectrometer operates with effective DNP sample volumes of 30 micro-litres in induction mode. This additionally allows direct in-situ EPR detection during high-power microwave pulses under DNP experimental conditions, as well as permitting a range of more conventional EPR experiments with very high concentration sensitivity^56^. It can also operate with cw illumination at lower power levels of 200 mW.

Using a pulsed inversion scheme we demonstrate experimental static enhancements for mono-radicals as high as 340 for 4-amino TEMPO, and more than 230 for b-PyTol at 60 K. At 100 K an enhancement of 260 is achieved with the biradical AMUPol. Moreover these gains are achieved using relatively low average powers of a few hundred mW (see Supplementary Table 1), and fast build-up times (time constants as short as 3 s at 60 K), for both mono-radicals and biradicals. These are some of the highest static cross-effect DNP enhancements achieved with large-volume non-resonant sample holders. The temperature dependence of the enhancement for different radicals also provides additional insight into fundamental DNP mechanisms.

## Results and discussion

### Cross-effect DNP with 50 mM 4-amino TEMPO in “DNP juice”

All the work was performed at 94 GHz (3.35 T) using a home-built broadband (1 GHz) high-power (1.3 kW) pulsed EPR spectrometer (HiPER) that has been adapted for DNP measurements. This is described further in the methods section.

**Fig. 1:**
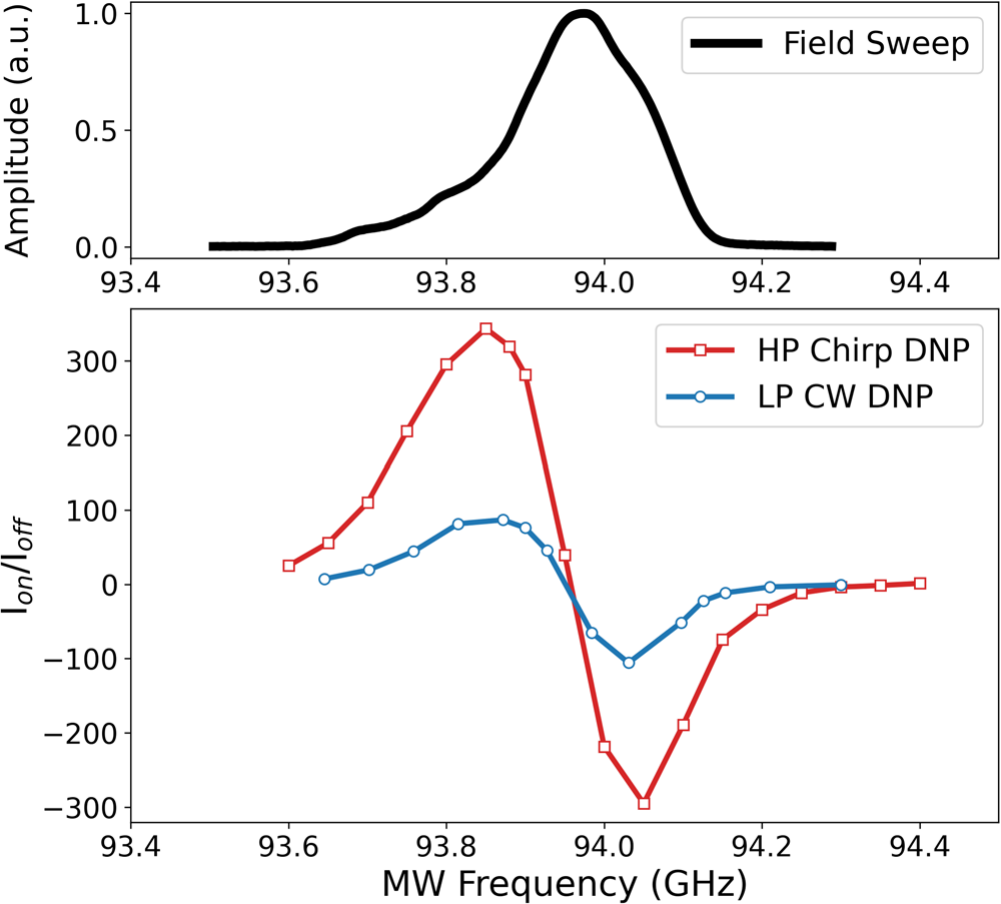
Dynamic nuclear polarisation (DNP) enhancement versus microwave frequency and EPR spectrum. Dynamic nuclear polarisation enhancement versus chirp centre frequency for 50 mM 4-amino TEMPO in DNP juice at 60 K (bottom) with low power cw excitation (blue) and high-power chirped inversion pulses (red). Roughly three to four times larger enhancements are achieved when using high-power (υ_1e_ ~ 42 MHz) inverting chirped pulses (estimated average power ~300 mW at the sample) compared to lower power (200 mW) cw excitation. The curves show the classic characteristics of DNP enhancement from the cross-effect, with possible contributions from thermal mixing. The same WURST chirped pulses were used in all measurements with just the centre frequency varying. The EPR spectrum as a function of frequency is shown in black (top).

Figure 1 shows DNP enhancement as a function of microwave centre frequency using the mono-radical polarising agent 4-amino TEMPO at 50 mM concentration in 50 μL of “DNP juice” at 60 K at a field of ~ 3.35 T. DNP juice consists of d_8_-glycerol/D_2_O/ H_2_O in the volume ratio 60:30:10 and is known to provide high proton DNP enhancements. The red trace shows DNP following 10 s of a stream of chirped microwave inversion pulses, where the pulse duration, frequency range, pulse shape, repetition rate and temperature were all carefully optimised for DNP. Wideband uniform rate smooth truncation (WURST) pulses were used with a chirp width of 270 MHz and a length of 120 ns. The peak power at the sample was ~ 600 W, and the optimised repetition frequency was 4 kHz at 60 K, for an average power of ~300 mW at the sample. The blue trace shows DNP enhancement after 60 s irradiation using cw radiation at a power level of ~200 mW at the sample.

Enhancements exceed 340 at 60 K for an optimised chirp inversion pulse (B_1eff_ ~ 15 G, υ_1_ ~ 42 MHz), compared to an enhancement of just over 100 using low power cw excitation (B_1eff_ ~ 0.2 G, υ_1_ ~ 0.56 MHz). Both the cw and chirped pulse DNP enhancement curves are characteristic of the cross-effect (although we cannot rule out additional contributions from thermal mixing).

This result shows that large static enhancements are possible using pulsed chirped excitation with fast polarisation build-up times at relatively low average powers. Compared to conventional single-frequency inversion pulses, chirped inversion pulses offer excitation over larger bandwidths and better compensate for B_1_ inhomogeneity. This was also validated by DNP experiments that compared short single-frequency rectangular or Hamming sinc pulses to chirped pulses with comparable bandwidths (also see Supplementary Fig. 1).

### Temperature dependence of DNP efficiency

**Fig. 2:**
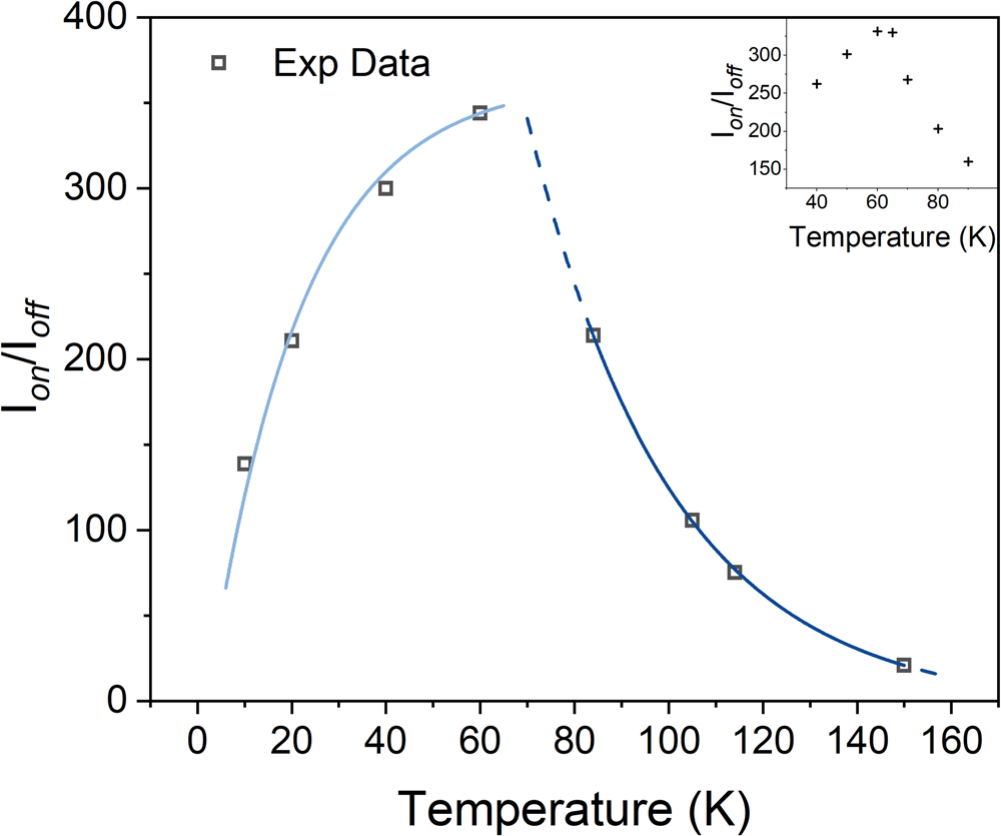
DNP enhancement as a function of temperature. DNP enhancement as a function of temperature, using optimised chirped inversion pulses on 4-amino TEMPO at 50 mM concentration in DNP juice. The blue lines show a fit to the experimental data points to indicate the trend. A dramatic change in enhancement is seen around 65 K where molecular motion and methyl rotation is expected to become significant. The inset plot shows more data points around this transition temperature, and were acquired during a separate experiment (on the same sample).

Figure 2 shows the DNP enhancement for 4-amino TEMPO as a function of sample holder temperature from 6 K to 150 K, indicating a peak close to 65 K, as shown in the inset figure. Below 65 K, we associate the rapid increase in enhancement with temperature to a shorter T_1e_, which allows a faster repetition rate and reduces the effect of spectral diffusion. Above 65 K, we associate the decrease in enhancement with the onset of methyl rotation and librational motion. We presume either acts as a local nuclear polarisation sink that reduces the effective DNP rate^39^ or more directly dephases the cross-effect transition. In contrast, polarising agents without methyl groups (shown later) show a more gradual decrease in enhancement with temperature. We are not aware that this temperature dependence has been observed before, but we note that there have been surprisingly few DNP studies in the temperature range 30 K–70 K^57^, especially at elevated power levels.

Chirp pulse lengths and frequency scan ranges were optimised at each temperature, and optimal pulse lengths found to be slightly longer at lower temperatures. Chirped pulses were found to give higher enhancements compared to cw illumination except at very low temperatures (<20 K), where even low-power cw radiation provided comparable results (see Supplementary Figure 1). This is attributed to the long T_1e_ found at low temperatures that allows time for spectral diffusion to reduce the steady-state polarisation gradient.

### Build-up time

**Fig. 3:**
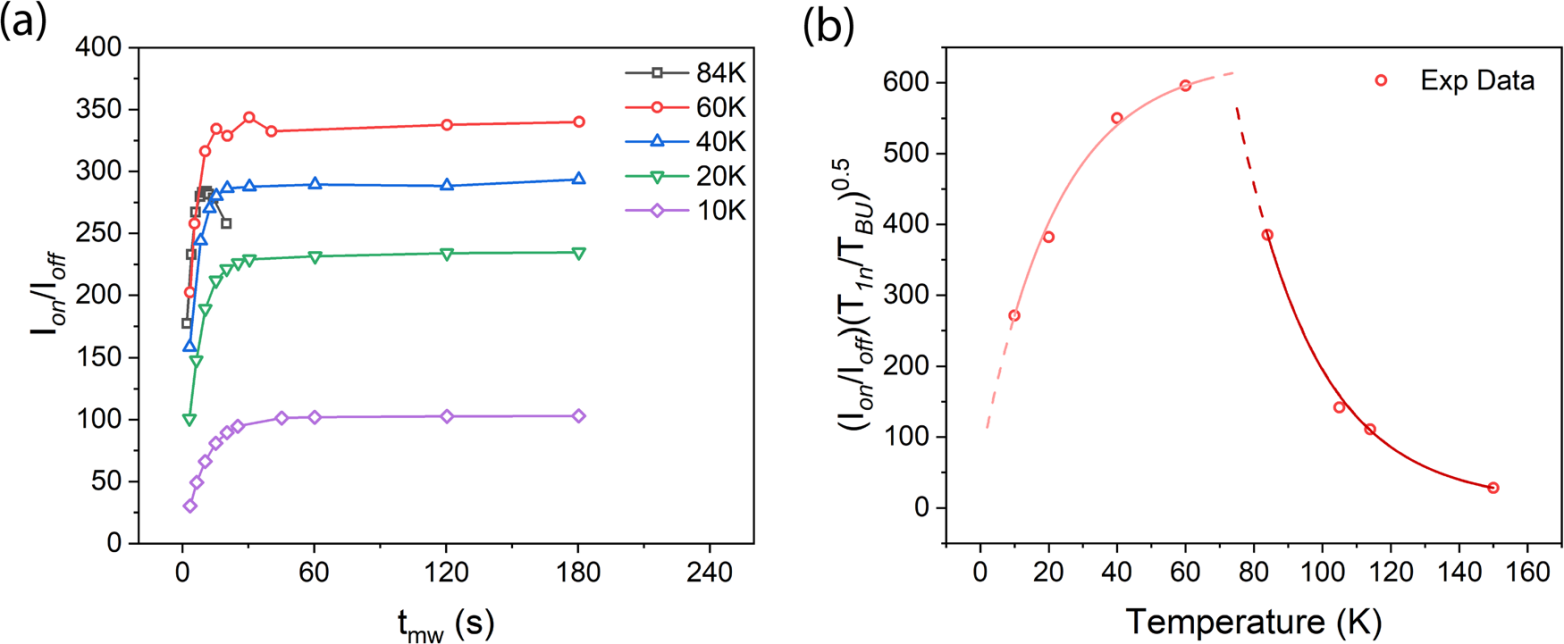
DNP build-up time as function of temperature. **a** Graphs showing the DNP build-up at different temperatures for 50 mM 4-amino TEMPO in DNP juice. The apparent drop in enhancement for the 80 K (black) trace at longer illumination is due to heating. **b** Practical DNP enhancement as a function of temperature taking into account the sensitivity increase due to a short T_BU_ with respect to T_1n_. The red lines are a fit to the experimental data points as a visual guide to the changing trend.

For practical DNP applications, the polarisation build-up time is also extremely important. Figure 3a shows the DNP build-up as a function of temperature, under conditions where the pulse parameters have been optimised at each temperature. For temperatures up to 60 K we obtained the build-up time T_BU_ by fitting the DNP data to the function 1-exp(-t/T_BU_). Above 65 K heating effects become more apparent and the DNP enhancement was observed to drop with increasing microwave illumination duration. For these cases T_BU_ was defined as the time needed to reach ~63% of maximum DNP enhancement. Values of T_BU_ are shown in Table 1 in comparison to measured values for T_1n_ (in the presence of the free radical), as well as the time T_90%BU_ to reach 90% of the maximum enhancement.

**Table 1:** DNP enhancement, build-up time and T_1n_ at different temperatures.

Temperature(K)	T_BU_(s)	T_90%BU_(s)	T_1n_(s)	I_on_/I_off_	(I_on_/I_off_)(T_1n_/T_BU_)^0.5^
150	0.6	2.7	1.1	21	28
114	1.5	4.1	3.3	75	111
105	2	5.1	3.6	106	142
84	2	5.3	6.5	214	385
60	2.5	8.5	7.5	344	596
40	4.3	11.1	14.5	300	550
20	6.0	15.7	19.8	210	382
10	9.9	23.1	37.8	139	271

It should be noted that the build-up time is always shorter than the T_1n_ relaxation time for protons (in the presence of radicals) particularly at lower temperatures. Thus, the recycle delay time for NMR with DNP can be shorter than the recycle delay time for NMR alone. It is therefore possible to average faster per unit time with DNP and increase effective SNR. A fairer comparison of the practical DNP enhancement is given by $({I}_{on}/{I}_{off})\sqrt{{T}_{1n}/{T}_{BU}}$. This is shown in Fig. 3b for T_1n_ measured in the presence of radicals. In the absence of free radical polarising agents T_1n_ will of course be much longer, and the effective sensitivity gains considerably larger.

### Optimal repetition rate

The optimal pulse repetition rate is expected to be strongly related to the inverse of the electron spin-lattice relaxation time T_1e_, at a given temperature. If the repetition rate is slow compared to 1/T_1e_, the number of effective electron-electron-proton flip-flop events is reduced. This is important as, for full nuclear polarisation, a single electron needs to polarise thousands of nuclei on the timescale of T_1n_. On the other hand, if the rate is too fast compared to 1/T_1e_, the polarisation gradient will reduce until eventually the electron spin system saturates. There may also be insufficient time for effective nuclear spin diffusion before the next pulse. Thus the optimum repetition rate will be a balance between all these effects.

**Fig. 4:**
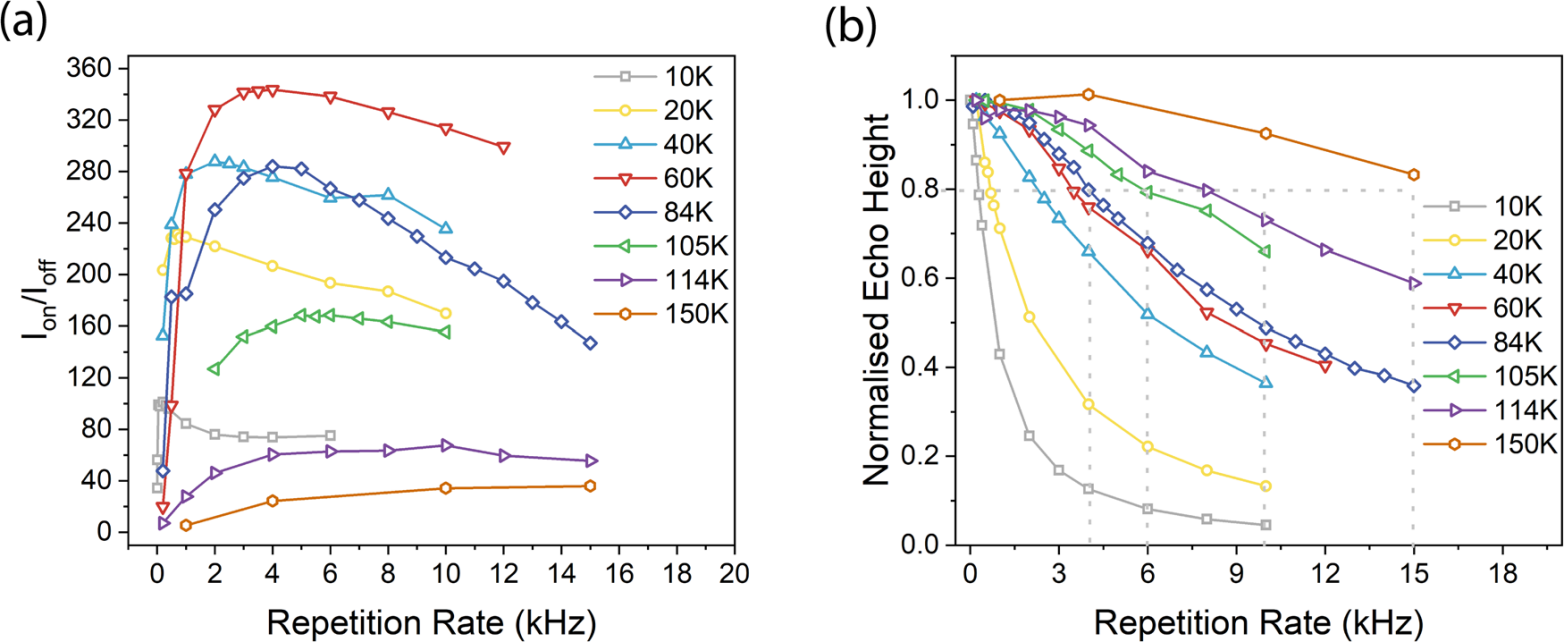
DNP enhancement and EPR echo height correlation with pulse repetition rate. **a** DNP enhancement as a function of repetition rate. **b** EPR echo height as a function of repetition rate, at line centre. To a good approximation the optimal repetition rate for DNP coincides with the repetition rate (1/T_80%SRT_) which reduces the spin echo to ~ 80 % of its maximal value.

Figure 4a shows DNP enhancement as a function of repetition rate. The rate that gave the largest DNP enhancement was found to strongly correlate with the recovery rate for an EPR Hahn echo experiment using soft 100 ns excitation pulses. Specifically, the optimal repetition rate for DNP was found at 1/T_80%SRT_, where T_80%SRT_ is the shot repetition time (SRT) that reduces the amplitude of the Hahn echo to 80% of the echo height obtained at slow repetition rates. This is indicated in Fig. 4b. It should be noted that the recovery rate 1/T_R_ associated with narrowband excitation of a broad line at 50 mM concentration, is expected to be dominated by spectral diffusion at low temperatures. On the other hand, the recovery rate associated with the wideband inversion pulse is expected to be dominated by the slower longitudinal relaxation rate 1/T_1e_, at least away from the excitation band edges. A table showing values of T_M_, T_R_ and T_80%SRT_ as a function of temperature is shown in Supplementary Table 2. T_M_ and T_R_ values as a function of temperature are also plotted in Supplementary Fig. 2.

At 150 K, the optimal repetition rate of ~15 kHz is still well below the maximum modulation rate of 80 kHz of the pulsed EIKA used in these experiments.

### Polarisation gradient and spectral diffusion probed via ELDOR experiments

To obtain further insight into the optimal repetition rate, temperature-dependent electron double resonance (ELDOR) studies were undertaken to obtain a snapshot of the magnetisation after the inversion pulse at the optimal repetition frequencies.

**Fig. 5:**
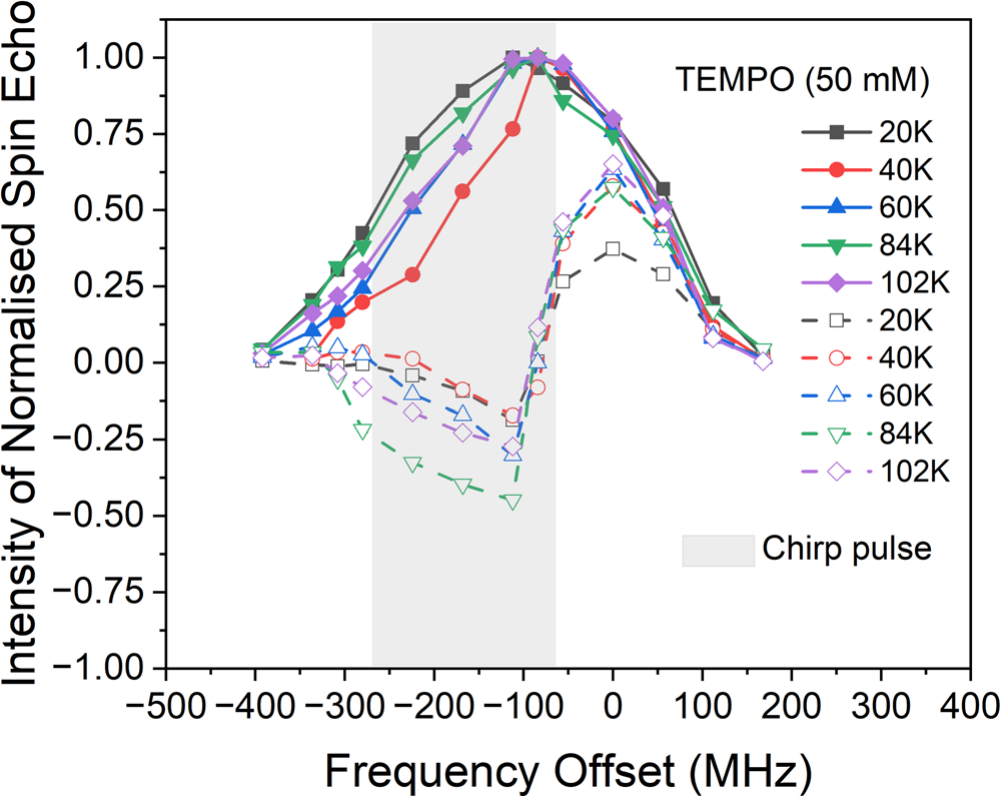
ELDOR measurements as a function of temperature. Temperature-dependent ELDOR measurements on 4-amino TEMPO (50 mM) showing the effectiveness of the inversion pulse under steady-state conditions at optimal repetition rates. The solid lines show normalised field swept echo heights at different temperatures. The dashed lines show normalised field swept echoes taken 4 μs after the chirp pulse at different temperatures. It should be noted that all data were taken at repetition rates optimised for DNP, and all data were normalised to the peak of the spectrum, at a given temperature.

Figure 5 shows the electron spin echo amplitude of an observer Hahn echo sequence measured across the line: without chirped microwave excitation (solid curves); and 4 µs after chirped microwave excitation (dashed curves). The pulse sequence is shown in Fig. 10 in the Methods section. In this experiment the observer frequency was kept constant while the magnetic field and centre frequency of the chirp pulse were stepped synchronously. The grey-shaded area in the figure denotes the frequency range of the chirp pulse that provides the largest positive DNP enhancement at 60 K. The optimal excitation bandwidth of ~230 MHz was found to vary slightly with temperature, but the optimal start (or end) frequency near the centre of the line was found to be temperature invariant. DNP enhancement was also found to be independent of the direction of the chirp pulse.

The graphs have been normalised to the observed spin echo height at the peak of the spectrum without the chirped pulse. The same data normalised to the echo height at each frequency without the chirp pulses are shown in the Supplementary Fig. 3. Soft π/2 and π observer pulses of length 100 ns were used to reduce the effects of instantaneous diffusion. Note, the data shown are measured at the repetition frequency that optimises DNP at each temperature. Without the chirp pulse, the ELDOR spectrum is essentially a distorted field swept EPR spectrum. In the presence of the chirp pulse, the ELDOR spectrum gives a measure of the true inversion efficiency and indicates the effects of spectral diffusion. It should be noted that less distortion in the EPR spectra, and >90% inversion efficiency is achieved by using repetition rates much slower than 1/T_1e_.

There are two key observations from Fig. 5. Firstly, the optimal polarisation gradient reduces at the lowest and highest temperatures, indicating the optimal repetition rate becomes fast relative to 1/T_1e_. Secondly, the temperature (60 K) where the largest DNP enhancement is obtained is lower than the temperature (84 K) where the largest steady state polarisation gradient is observed.

The significant decrease in observed polarisation gradient at low temperatures is consistent with 1/T_1e_ becoming slow relative to the spectral diffusion rate. Analogous arguments, relating to the deleterious effects of spectral diffusion, have been made with DNP experiments using saturating radiation^30^. The loss of polarisation gradient at low temperatures is consistent with observations from several other experiments^58–61^.

The decrease in optimal polarisation gradient, observed at higher temperatures, indicates that other “fast” mechanisms start to become important, possibly associated with the onset of molecular librational motion, as discussed later.

### Enhancement as a function of peak and average power and heating effects

**Fig. 6:**
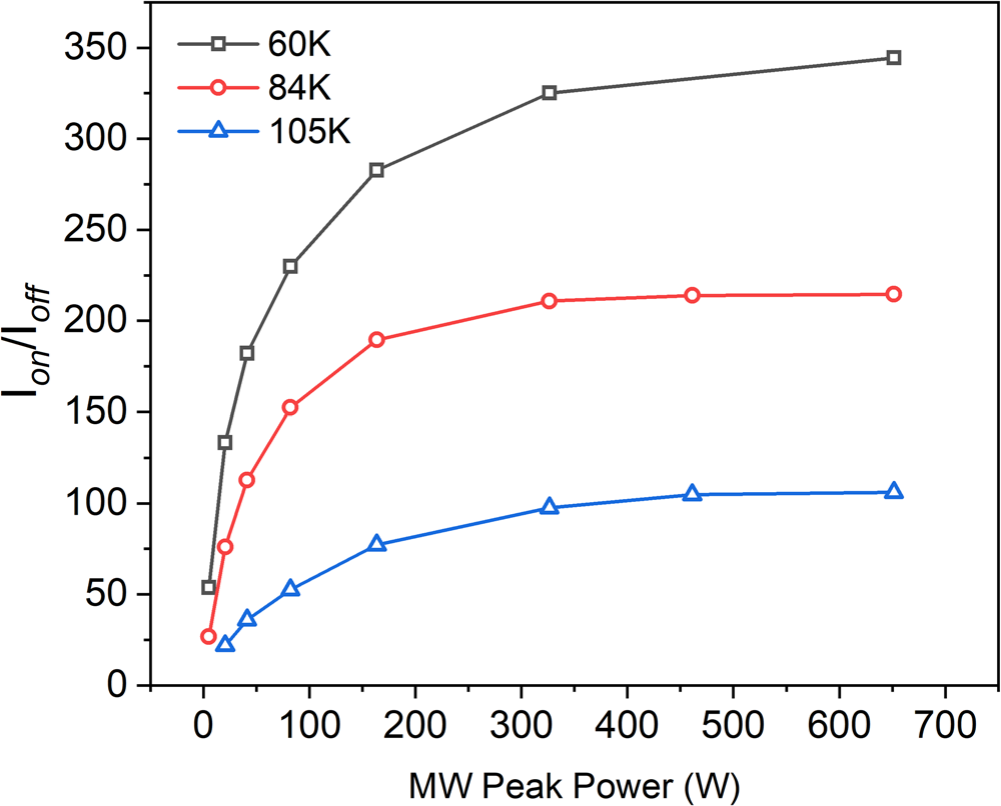
DNP enhancement as a function of microwave peak power. DNP enhancement as a function of microwave peak power at the sample (50 mM 4-amino TEMPO) at three different temperatures. Note the estimated power at the sample is around half the power available at the EIKA output.

Figure 6 shows DNP enhancement against peak power level for three different temperatures, 60 K, 84 K and 105 K, under experimental conditions that are optimal at the maximum power level. In general, the results indicate we are operating under conditions where we are not limited by the available peak power over this temperature range. The power dependence was not measured at lower temperatures, but we note that enhancements were not large at low temperatures and similar to low power cw excitation (see Supplementary Table 1).

A figure of merit for chirped pulses is the critical adiabacity factor, Q_crit_, given by ${Q}_{crit}=2\pi {{\nu }_{1}}^{2}\varDelta t/\varDelta f$ for a linear chirp where ${\nu }_{1}=\gamma {B}_{1}/2\pi$ and B_1_ is the microwave magnetic field at the sample^62^. A Q_crit_ of 0.44 would correspond to a π/2 pulse whereas Q_crit_ > 2 is expected to have an inversion efficiency >95%.

In these experiments the sample holder is essentially a shorted waveguide (see Fig. 8 in the methods section) leading to a significant variation in B_1_ field along the length of the sample. The expected variation in B_1_ is shown in Supplementary Figure 4, derived from simulations using CST Microwave Studio Suite. It is also possible to estimate an effective average B_1_ ~ 15 G (*υ*_1_ ~ 42 MHz) from the experimental length of the effective π/2 pulse length (6 ns), which is consistent with the CST simulations. For a typical frequency chirp of 270 MHz of length 120 ns, this gives an effective typical Q_crit_ of around 4.75. Thus we expect to have high inversion efficiency over much of the sample and we are not meaningfully limited by peak power.

A more practical limitation is the average power handling of the system. In principle the EIKA (designed for radar applications) can deliver an average power of 100 W, but in practice average powers are limited both by sample heating and the power handling capabilities of a waveguide isolator (10 W) used in our system to protect the EIKA. Thus, in our current configuration, maximum average power levels are usually kept below 3 W, to maintain a significant safety margin, and to extend the life of the tube. This was not a significant limitation in our measurements and the average power is comparable to, or less than, the average power provided by cw gyrotrons commonly used for solid-state DNP at higher frequencies. However, we note that higher average powers are likely to be necessary for polarising agents that utilise the solid effect^63^, or have shorter relaxation times such as many transition metals.

### DNP enhancements with other polarising agents

It is interesting that the large drop in DNP enhancement observed above 65 K for 4-amino TEMPO shown in Fig. 2 coincides with the well-known onset of thermally activated motion and methyl rotation in many nitroxides^64^. Methyl rotation in particular is known to cause a significant decrease in phase memory time (T_m_) above 65 K, due to modulation of hyperfine couplings. This is the reason, for example, why most nitroxide PELDOR measurements are made in the temperature range 50–60 K. The onset of thermal molecular motion can also modulate dipolar couplings between spins, leading to additional relaxation processes, causing a reduction in T_m_^65^. We also note that the most efficient biradical polarising agents currently used in MAS DNP have no methyl groups and are designed to be bulky and more rigid, usually with the stated aim of lengthening T_1e_ and T_m_ to make them easier to saturate^24,26^. However, the measurements described in this paper are not significantly power limited. Thus one can speculate that at elevated temperatures, it is thermal motion which plays a more direct role in interfering with coherent polarisation transfer.

To further investigate the rapid change in DNP enhancement with temperature, we also measured DNP enhancements with two other water-soluble polarising agents and correlated their DNP performance with associated changes in phase memory time T_m_. These included the mono-radical b-PyTol^39^ at 50 mM concentration, and the biradical AMUPol^25^ at 10 mM and 17 mM concentrations (20 mM and 34 mM spins). b-PyTol does not contain any methyl group. AMUPol does contain one methyl group, but it is not in close proximity to the nitroxide. In all experiments the solvent was “DNP juice”, d_8_-glycerol/D_2_O/ H_2_O in the ratio 60:30:10 except for the 17 mM AMUPol sample, which was in the ratio 60/34/6. In all cases, very similar experimental conditions were found to optimise enhancements.

**Fig. 7:**
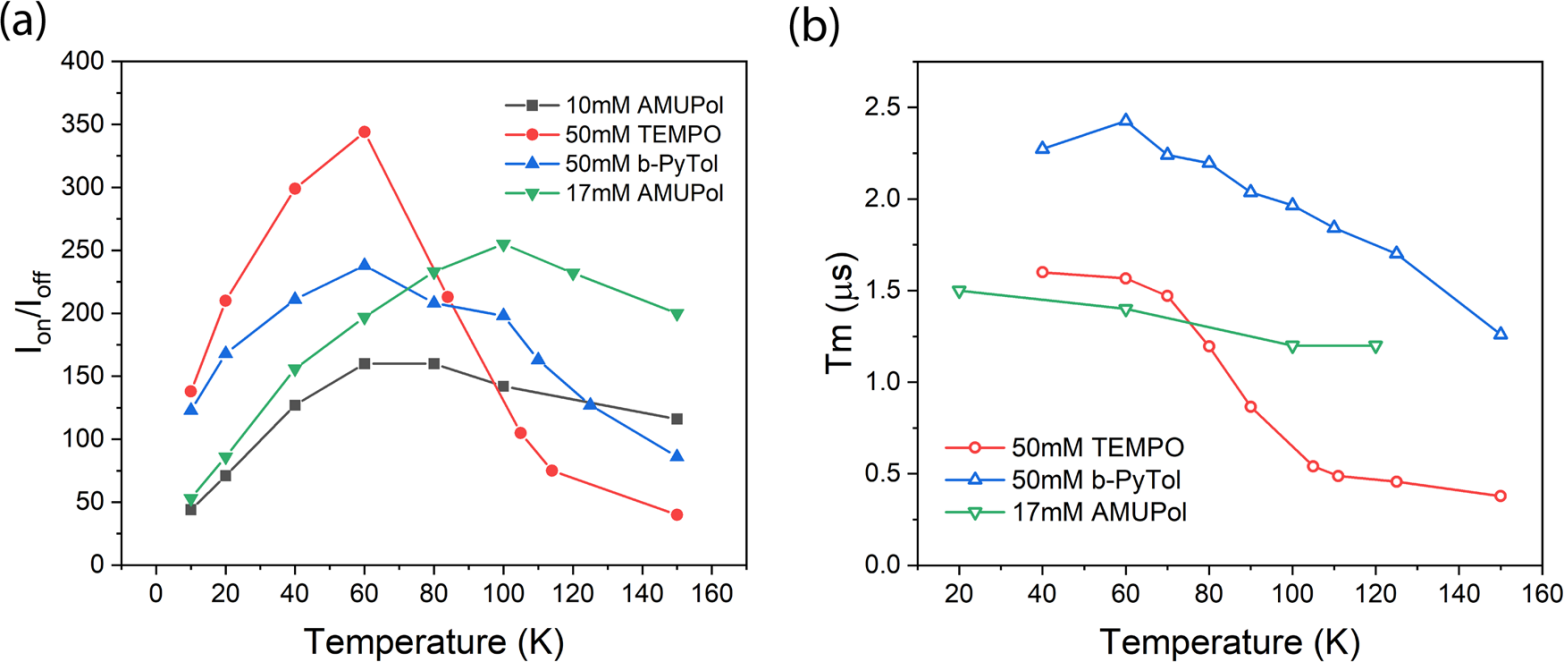
DNP enhancements and phase memory time as a function of temperature. **a** DNP enhancement as a function of temperature for the mono-radicals 4-amino TEMPO (red) and b-PyTol (blue) both at 50 mM concentration and the biradical AMUPol at 10 mM concentration (black) and 17 mM concentration (green). **b** electron phase memory time T_m_ as a function of temperature for the different polarising agents.

DNP enhancements and phase memory time for these polarising agents, as a function of temperature, are shown in Fig. 7a. and Fig. 7b. respectively.

In contrast to 4-amino TEMPO where enhancement drops rapidly above 65 K, both b-PyTol and AMUPol, without local methyl groups, offer much higher enhancements near 100 K, at temperatures more relevant for MAS. The effective drop in cross-effect DNP rate for 4-amino TEMPO also coincides with decreasing T_m_. Thus, in these experiments we take a significant reduction in T_m_ above 65 K as a proxy for reduced DNP efficiency. The smallest reduction in T_m_ with temperature was observed for AMUPol, which we attribute to the larger molecular weight reducing thermal motion of the paramagnetic centre. This correlates with the much higher efficiencies observed at higher temperatures for AMUPol, even peaking at 100 K for the 17 mM sample.

Below 65 K, at low nitroxide concentrations, it is well known that T_m_ relaxation is dominated by proton spin diffusion. However, at high radical concentration, the phase memory time is expected to be much shorter and dominated by flip-flop rates of electrons^64^. In Supplementary Fig. 5 we show T_m_ rapidly decreasing as a function of 4-amino TEMPO concentration taken at Q-band. Thus T_m_ below 65 K might be expected to partially correlate with the strength of the cross-effect and be relatively independent of temperature, except at very low temperatures when full electron polarisation is approached^33^. Therefore, T_m_ in this temperature range should be related to the probability of finding electron spin pairs at suitable frequency offsets. It should be noted that the measured low-temperature phase memory times of: ~1.1 μs at 34 GHz (Supplementary Fig. 5); ~1.6 μs at 94 GHz (Supplementary Fig. 2); and 4 μs at 250 GHz^32, 33^, for 50 mM 4-amino TEMPO, are all consistent with differences in linewidth at the different frequencies.

During the reviewing process, one reviewer suggested using deuterated TEMPO to prove that methyl groups were detrimental to DNP and as a potential means to increase enhancements at higher temperatures. We note this strategy has been used before with TOTAPOL at 180 GHz with significant enhancements observed at 110 K^66^. This is currently the subject of ongoing work.

### Discussion and conclusions

To summarise, this study shows that large cross-effect DNP enhancements with fast build-up times can be achieved using chirped inversion pulses at 94 GHz at optimised repetition rates with large volume samples. These are some of the largest static cross-effect DNP enhancements demonstrated using nitroxides. This is promising as DNP enhancements with MAS are typically observed to be several times larger than those achieved with static configurations. These large gains have also been achieved with polarising agents not previously known for their high DNP efficiency. This may have implications for the choice of DNP polarising agents in reducing environments^39^.

These results can be compared to: Kaminker et al. who achieved an impressive cross-effect DNP enhancement of 500 at low temperatures (4 K) using cw frequency swept DNP with 10 mM AMUPol^46^. A similar enhancement (>380) has been achieved by Tan et al. at 80 K using the adiabatic solid effect at 1.2 T with a resonator probe^67^. More recently, and during revisions for this paper, Griffin’s group described their very promising work using the adiabatic solid effect with chirp pulses using the same HiPER setup at the US National High Magnetic Field Lab (NHMFL) in Florida, where they achieved a spectacular DNP enhancement ~500^68^.

However, pulsed inversion schemes using the cross effect should require lower average power levels, have faster build-up times, and potentially offer better scaling to higher frequencies. At higher fields, at least for solid-state proton DNP, it should be technically easier to excite allowed transitions over broad frequency ranges with nitroxides, relative to the solid effect which requires efficient excitation of forbidden transitions in narrow-line systems^44,51,69–71^.

Relative to saturation pulses, inversion pulses maximise the initial polarisation gradient across a nitroxide line, whilst chirp pulses optimise the excitation bandwidth and naturally compensate for B_1_ inhomogeneity across the sample. The repetition rate provides another important control parameter, where the optimum rate is a balance between allowing the electron polarisation to recover (via T_1e_ processes) and limiting the time for spectral diffusion that can limit the steady state polarisation gradient. Thus, a faster T_1e_ at higher temperatures is seen to be highly beneficial in improving DNP efficiency, at least up until a temperature of around 60 K. At higher temperatures, it would appear important to reduce thermally activated processes that would normally contribute to decreases in T_m_, and to avoid local methyl groups. This is consistent with current molecular design strategies known to optimise polarising agents^24,26^.

In the steady state the gain due to DNP will also be balanced by the polarisation loss due to T_1n_, suggesting that a variable repetition rate strategy, to further speed up DNP polarisation, may be worthy of investigation. However, the rate of polarisation is already fast compared to T_1n_ and may already lend itself to rapid dissolution strategies for both NMR and MRI, at more elevated temperatures than used in conventional dissolution DNP experiments^72,73^.

All three polarising agents have essentially the same optimal repetition rates at a given temperature, suggesting common limiting relaxation mechanisms. It is also interesting that the optimum repetition rate was found to reduce the polarisation gradient above 70 K, despite the faster T_1e._ This is the subject of ongoing investigations using time-dependent ELDOR, where we emphasise the importance and usefulness of simultaneous EPR characterisation of DNP polarising agents over broad temperature ranges, at high power levels.

Two reviewers also suggested there might be an additional contribution due to thermal mixing, which requires strong dipolar coupling. Indeed, we cannot rule this out, especially for the mono-radicals. The results are not inconsistent with theoretical models presented by Wenckebach^12,13^, and recent work by Equbal et al. ^30,74^, where small molecules are required to ensure there is a statistical probability that some spin pairs are close enough to have a dipolar coupling frequency that is comparable to the nuclear frequency. Previous studies have also observed that thermal mixing DNP can coexist with other DNP mechanisms in solid-state DNP^75,76^. This might partly explain the higher enhancements achieved with both b-PyTol and 4-amino TEMPO relative to previous measurements at higher fields (where nuclear frequencies are higher relative to the dipolar coupling). It might also explain why the onset of thermal motion, modulating dipolar and hyperfine couplings, would be more deleterious for the mono-radicals. This is currently the subject of further investigation.

It would of course also be interesting to investigate concentration dependence, and use similar pulsed techniques to evaluate DNP enhancements with some of the new optimised biradicals such as HyTek2^28^, TekPol^26^, O-MbPyTol^39^, and cAsymPol-POK^27^. These polarising agents have achieved some of the highest reported MAS DNP enhancements using cw saturation techniques. It would also be interesting to evaluate the importance of first deoxygenating the samples to increase nuclear T_1n_^68^.

Finally, we believe this work provides extra motivation for the development of high-power amplifiers at much higher frequencies than 94 GHz. Today, klystron pulse amplifiers, of the type used in this study, suffer a significant drop-off in efficiency above 94 GHz. However, in the future, gyro-amplifiers with low dispersion waveguides are expected to offer much improved scaling to higher frequencies with high peak power levels over extended bandwidths^77^. If these amplifiers can be scaled to higher frequencies they are likely to satisfy the main technical requirements for pulsed cross-effect at high fields. We believe that the results described in this paper support such developments.

## Methods

### Sample description and preparation

4-amino TEMPO (4-amino-2,2,6,6-tetramethyl-piperidine-1-oxyl) was purchased from Sigma-Aldrich, b-PyTol was provided by the laboratory of Olivier Ouari in Marseille (CNRS) and AMUPol was provided by the laboratory of Walter Köckenberger at University of Nottingham. Initial experiments focussed on 4-amino TEMPO, due to its high water solubility. All samples were dissolved in a mixture of d_8_-glycerol/D_2_O/H_2_O, in volume ratios referred to in the text. It should be noted that no attempt was made to deoxygenate the samples before measurement.

**Fig. 8:**
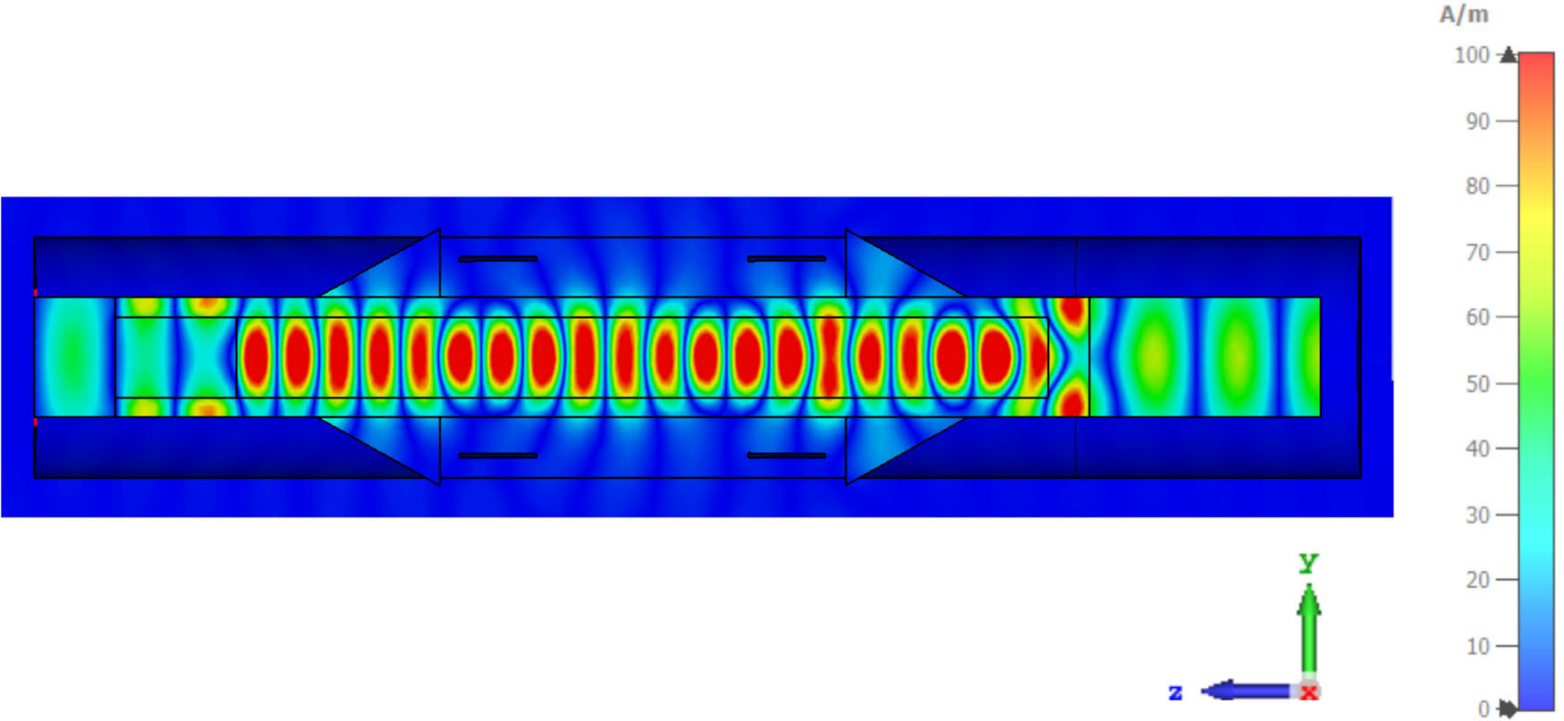
Simulated B_1e_ distribution across the sample. Simulated B_1e_ distribution in the 3 mm diameter microwave transmission line, containing the sample tube and sample, also showing the TE_11_ mode microwave transitions and indicating the position of the saddle coil. The peak conversion factor in the sample is 160 A/m per root watt, and the average ~ 50 A/m per root watt. (Simulated using CST Microwave Studio Suite).

All samples were measured in a FEP (fluorinated ethylene propylene) tube with 2 mm inner diameter and 3 mm outer diameter. The effective active volume in the DNP experiment was around 30 μL, although larger sample volumes were used in the experiment, as indicated schematically in Fig. 8. The samples in the FEP tube were first flash-frozen in liquid nitrogen prior to loading into a pre-cooled sample holder cartridge. The cartridge is then pushed into a spring-loaded mount, which is part of a larger microwave transmission line assembly consisting of an optimised feedhorn transition at the end of a long corrugated cylindrical pipe, contained within a flow cryostat. The microwave assembly is pre-cooled in a flow cryostat, typically to 150 K. It is designed to be rapidly removed from the flow cryostat, and reinserted, to allow cold loading of the sample cartridge. After sample loading the whole system is then cooled down to the desired experimental temperature, which ranges between 10 and 150 K.

The microwaves are terminated with an adjustable backshort below the sample, which reflects the radiation. This consists of a shallow roof mirror, which can be rotated or raised vertically using piezo-motors capable of operation at cryogenic temperatures. The roof mirror is usually adjusted to either maximise the cross-polar isolation, or maximise EPR (or DNP) signal, as the sample itself can act as a highly over-coupled low Q resonator.

### EPR/DNP spectrometer

The pulsed EPR spectrometer, adapted for this study, has been previously described^56^. It is known as HiPER and is home-built and uses an extended interaction klystron amplifier (EIKA) operating between 93.5 and 94.5 GHz with a peak pulse output power of around 1.3 kW. The spectrometer combines low loss quasi-optical techniques and microwave electronics to deliver around half of that power to a sample in a non-resonant sample-holder operating in induction mode with an effective B_1_ of around 15 G, and a conversion efficiency ~ 0.5 G /W^1/2^. Very little power is absorbed in the sample and most power is reflected back towards the transmitter. Isolation between the sample and transmitter is over 100 dB, facilitated by a combination of waveguide and quasi-optical isolators; the latter having negligible return loss. The EPR sample probe is essentially a shorted smooth circular waveguide capable of supporting two orthogonal linear polarisations^56^. The sample is excited by linearly polarised microwaves, and the orthogonal linear polarisation is used for EPR detection. This has the enormous advantage of providing considerable isolation between transmitter and detector thus eliminating the need for high-power receiver protection. This scheme offers very high concentration sensitivity for pulsed EPR measurements (around 50 x compared to commercial X-band EPR pulsed spectrometers) and the isolation between transmitter and detector can be sufficient to allow EPR measurements to be made simultaneously with high-power EPR excitation during DNP. The EPR spectrometer has recently been upgraded using an arbitrary waveform generator (AWG) (Keysight M8190A) that allows a wide variety of complex pulse sequences to be generated with variable amplitude, phase and frequency over 1 GHz bandwidths, with fully coherent detection. This facilitates the generation of broadband-shaped chirped pulses, as well as the (multi-frequency) ELDOR experiments described above in this paper. A more detailed description of the AWG system will be provided in a subsequent publication.

### DNP Sample holder

The sample is contained in a FEP tube of length 40 mm, 3 mm O.D. and 2 mm I.D., which is contained within a shorted single-mode circular waveguide transmission line of diameter 3 mm. FEP is preferred to quartz for low temperature measurements as it is found experimentally that it reduces the chance of sample fracturing due to differential thermal contraction. It also has a lower dielectric constant than quartz and so facilitates the “optical fibre” structure discussed below.

**Fig. 9:**
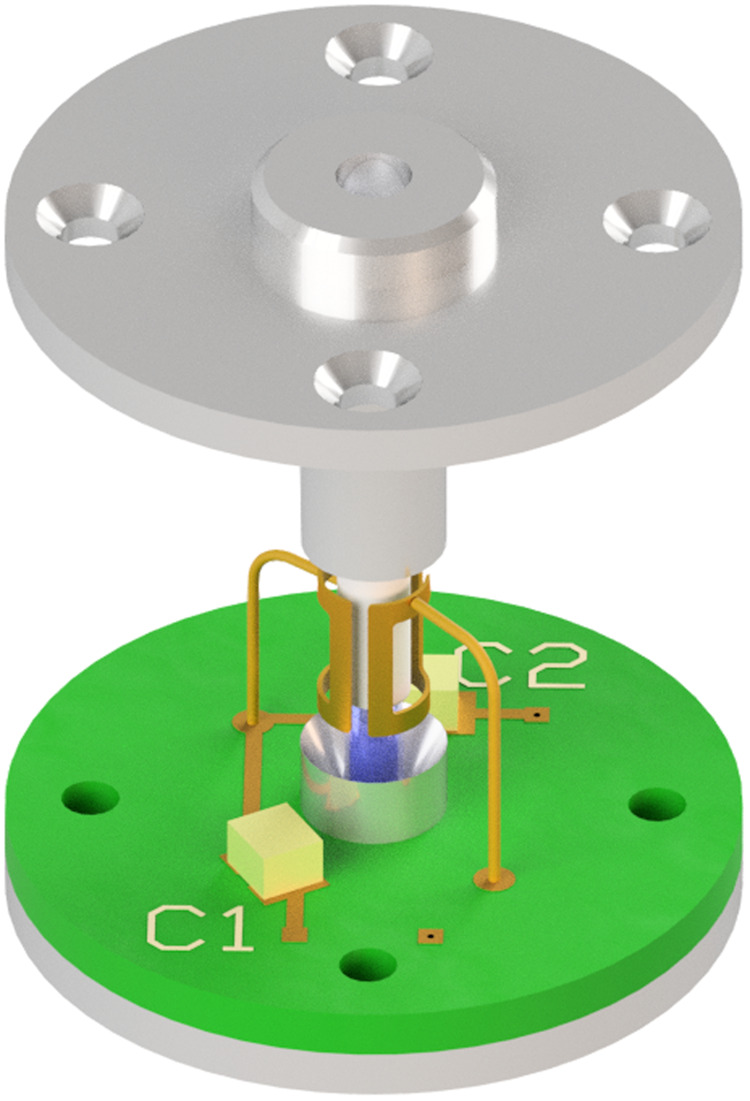
DNP probe and saddle coil experimental arrangement. Schematic drawing of the DNP probe showing the NMR saddle coil. High-power microwaves are transmitted down to a 3 mm diameter smooth waveguide that transitions into an open structure. The body of the holder is not shown for clarity. The FEP sample tube (3 mm O.D., 2 mm I.D.), with sample, acts as a guiding structure in this region. The transmission line then transits back into smooth guide where it is terminated by a shallow roof mirror that can be rotated and raised vertically via piezo-motors (not shown). This helps to match power into the sample and improve cross-polar isolation between excitation and detection channels.

The microwaves are transmitted to the sample holder via a 1.3 m long circular corrugated pipe and horn, located inside a flow cryostat, using the HE_11_ linearly polarised mode. The HE_11_ mode is then converted to the TE_11_ mode in smooth pipe via a suitable corrugated transition^78^. A smooth-walled taper transition (shown schematically in Fig. 8) couples the microwaves into an open “optical fibre” structure, consisting of the sample within the lower dielectric constant FEP tube, before transitioning back to a smooth-walled circular waveguide terminated by an adjustable short. Electromagnetic modelling using CST Microwave Studio Suite (Dassault Systèmes) showed that the smooth transition is necessary to avoid excitation of higher order modes in the sample in the fibre-optic section. Experiments showed that microwave B_1_ fields were similar to the enclosed waveguide structure typically used in standard EPR experiments, in agreement with simulations. The open waveguide structure allows the NMR signal to be easily detected using a home-built conventional tuned saddle coil, shown in Fig. 9.

The NMR coil was home-built and is cut from 0.1 mm thick copper film and bent into a saddle coil. The coil was gold-coated to prevent oxidisation. Cryogenic capacitors (American Technical Ceramics) were used to tune the NMR coil to resonance at ~143 MHz and match the circuit to 50 ohms. This allows the maximum effective power transfer from the RF amplifier to the saddle coil. The whole circuit is integrated on a circular PCB which fits into the sample probe as indicated in Fig. 9. During the tuning and matching process, all LC parameters of the coil and PCB strips were calculated from S_11_ measurements using a low-frequency VNA (Rohde & Schwarz ZNL6). A 3-term error correction model and de-embedding technique were used in the LC measurements. The measurement of inductance down to ~nH and capacitance down to ~pF allowed precise tuning and matching of the probe.

### NMR and DNP measurement description including reference measurements

All NMR and DNP experiments were recorded at ~3.35 T (W-band for EPR) with temperatures ranging from 10 to 150 K. The NMR acquisition was performed using a Tecmag Scout NMR system (Tecmag Inc.). The RF pulses, initially generated at low power, were first amplified by a 50 W RF amplifier (Electronic Navigation Industries, Model 550 L, 50 W RF power amplifier), and then directed by a transcoupler (NMR Service, 80–150 MHz) to the NMR probe. The returning RF signal from the probe is redirected by the transcoupler to a 34 dB preamplifier and finally to the NMR Scout receiver.

NMR reference experiments (without microwaves) were performed at different temperatures by recording either the integral or the amplitude of the free induction decay (FID) signal – where spin echoes also gave similar enhancements. FID detection was initiated after a delay of 7 μs after the excitation pulse, corresponding to the deadtime of the NMR spectrometer. At high temperature, 100 averages were typically required to achieve a good signal-to-noise for the reference signal, comparable to a heavily attenuated single-shot DNP-enhanced signal. The recycle delay for NMR measurements was set to be ~5T_1n_.

At low temperatures, the single-shot DNP enhanced NMR signal was so large that it could easily saturate the NMR spectrometer receiver system. To avoid this, a combination of three RF attenuators (Mini-circuits) (2 × 20 dB and 1 ×10 dB) were used to first attenuate the signal to be able to measure the FID signal under comparable NMR receiver gain settings. These attenuators were calibrated using a HP437B power meter and HP8481A power sensor and a HP8648 signal generator as the RF source. For the DNP enhancement comparison, all results used this calibration. The DNP enhancement was calculated as the ratio of the NMR FID (or spin echo) signal with and without the irradiating microwaves. Enhancements were checked for consistency by examining the maximum time domain signal, time domain trace integral, frequency domain resonance intensity and frequency domain integral. All methods gave similar DNP enhancements (+/- 10%).

A WURST chirp pulse was used in the DNP experiments unless otherwise noted. This pulse uses an amplitude window function of the form $1-\,\cos {|\pi t/\varDelta t\pi |}^{n}$ where t varies from 0 to Δt – the duration of the pulse, and where *n* = 50 was used in all experiments^79,80^. For each temperature, the pulse length, chirp frequency range and repetition rate were adjusted to obtain the optimal DNP enhancement. The optimal pulse length increased from 120 ns for 60 K to 300 ns for 10 K. The optimal chirp frequency range $\varDelta f$ changed from 93.66-93.93 GHz at 84 K to 93.72-93.93 GHz at 10 K, where interestingly $\varDelta f > {\omega }_{n}/2\pi$ (143 MHz). The optimal repetition rate of the microwave irradiation reduced from 15 kHz at 150 K to 0.2 kHz at 6 K. The chirp pulse illumination time increased from 5 s for 84 K to 23 s for 10 K. Immediately after microwave illumination one single RF π/2 pulse is applied through the NMR spectrometer and the ^1^H NMR FID is detected at ~143 MHz without averaging.

Heating effects were observed when high power microwave pulses were applied at higher repetition rates corresponding to average powers >1 W. The effects were particularly clear at higher temperatures (>65 K) where both DNP enhancements, and electron spin echo signals become strongly temperature dependent. Large variations in electron spin echo amplitude were observed over time-scales of tens of seconds during and after the application of high-power chirp pulses. These were clearly temperature related as the same effect was observed for chirp frequency ranges both inside and outside the nitroxide line. Above 70 K significant reductions in echo amplitude of sometimes more than 75% over periods of a minute were observed during excitation, recovering over similar periods when the microwaves were switched off. This effect introduces some uncertainty in determining the exact sample temperature. It was less clear whether this was significant at lower temperatures where dielectric losses are expected to be smaller^81^.

It should be noted that in these experiments both the sample and sample tube are effectively isolated from direct helium flow by the surrounding sample holder, and the thermal time constant associated with sample heating/cooling was typically much longer than the time constants associated with the DNP enhancement, especially at higher temperatures. Heating effects were mitigated by waiting for 1-2 mins before each new DNP measurement to make sure the sample had returned to thermal equilibrium.

The sample holder was designed in this way to avoid ice forming on the sample and piezo-motors during cold sample loading. However, it could be beneficial to improve sample cooling in future experiments. It should also be emphasized that the microwave dielectric losses are still relatively small, and the sample remains practically transparent at these frequencies (up to the glass transition temperature, usually well above 150 K, when microwave dielectric losses become very large). This is monitored by adjusting the position of the roof mirror beneath the sample and observing any resultant phase changes in return signals.

### DNP and ELDOR experiments

**Fig. 10:**
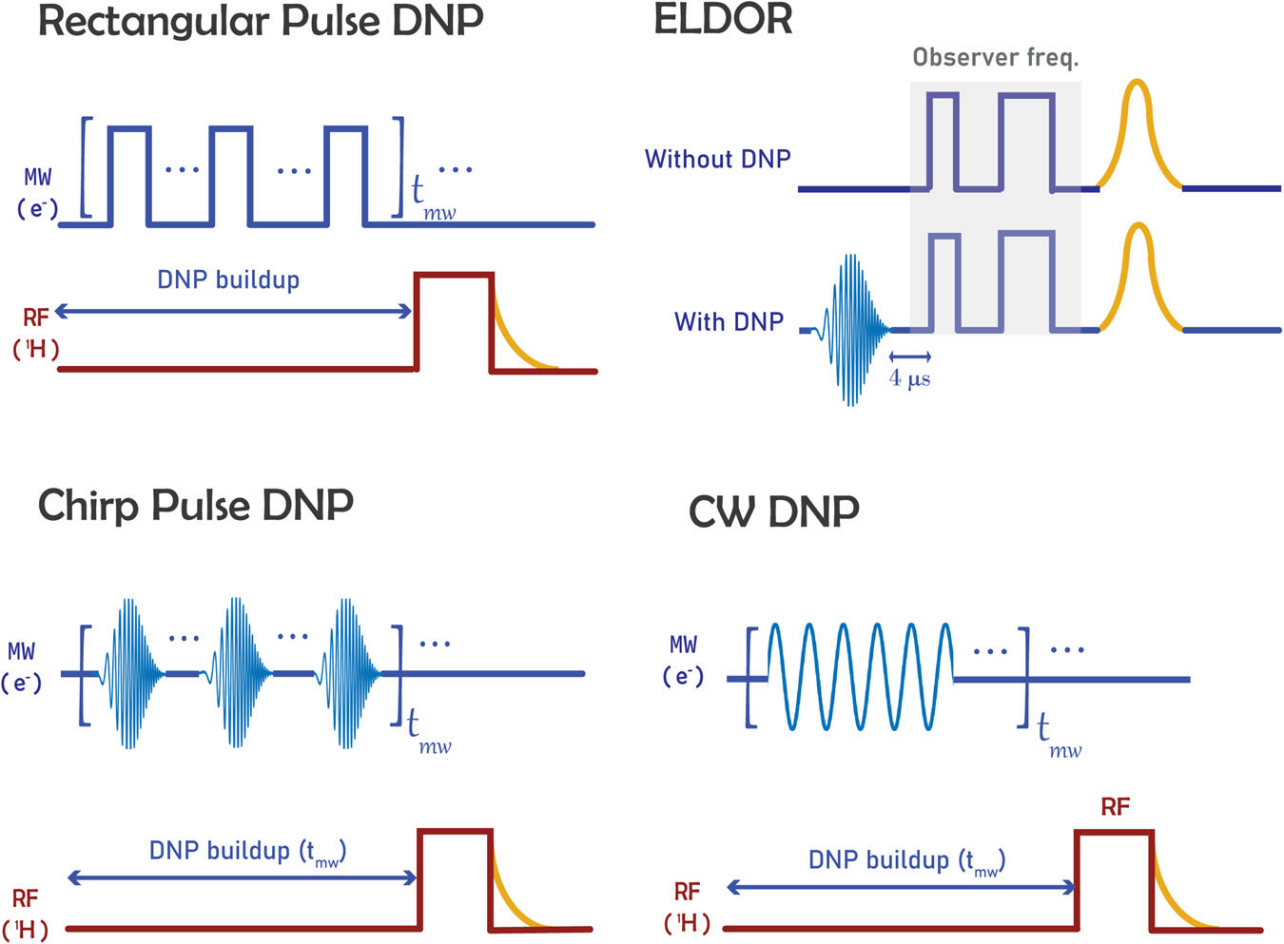
EPR and DNP pulse sequences. Schematic diagrams illustrating the pulse sequences used in the DNP and EPR experiments. For the DNP experiments either single frequency cw radiation or inverting pulsed radiation (rectangular or chirp) was used to create a polarisation gradient across the sample. DNP enhancements were taken by measuring the integral of the FID with and without microwave radiation. ELDOR experiments were used to give a qualitative indication of electron polarisation across the line as a function of pumping frequency.

For pulsed DNP experiments, microwave sweep parameters were first optimised (repetition rate, sweep range and pulse length), and then a single shot RF π/2 pulse of length (10 μs) was applied and the resulting FID signal was detected. All pulse sequencies used in this study are shown in Fig. 10. ELDOR measurements with the microwave inversion chirp pulse were normalised to ELDOR measurements without the chirp pulse. The detection pulses used a standard Hahn spin echo sequence at 94 GHz with rectangular pulses of 100 ns duration at reduced power levels. The electron spin echo pulse sequence was applied 4 μs after the chirp pulse. The time interval between π/2 and π pulses was set to 400 ns. Echo height is read after every 1024 averages at or above 84 K. At temperatures below 84 K fewer averages were required due to the improved signal-to-noise ratio. The experiment was run by keeping the observer frequency fixed at 94 GHz, whilst varying both the magnetic field and the frequency range of the chirp pulse.

### Relaxation measurement of electrons and protons

The decay of proton polarisation after 5 s of high-power chirp pulse DNP was measured and fitted with $A\exp (-x/{T}_{1n})$ to obtain the T_1n_ at different temperatures. The effective T_1e*_ was determined by exponentially fitting the electron spin echo height as a function of shot repetition time using $A(1-\exp (-SRT/{T}_{1e\ast }))$. It should be noted that this is an effective T_1e_ as it does not take into account spin diffusion. The electron phase memory time T_m_ was measured by two-pulse spin-echo decay experiments at relatively low power levels using long pulses (100 ns and 200 ns or longer) to reduce the effects of instantaneous diffusion.

### Supplementary information

Supplementary Information

## Data Availability

Extra experimental details can be found in the Supplementary Information. The main research data underpinning this publication can be accessed at 10.17630/769a70cb-e5d5-4078-9b21-96b6e3e887ef.
