# Supplementary material for: Large cross-effect dynamic nuclear polarisation enhancements with kilowatt inverting chirped pulses at 94 GHz
Source: Commun Chem. 2023 Aug 22;6:171. doi: 10.1038/s42004-023-00963-w (PMC10444895; doi:10.1038/s42004-023-00963-w)
Supplement: Supplementary file 1 — Supplementary Information [file 42004_2023_963_MOESM1_ESM.pdf]

Supplementary Information to:

"Large cross-effect dynamic nuclear polarisation enhancements with kilowatt inverting chirped pulses at 94 GHz"

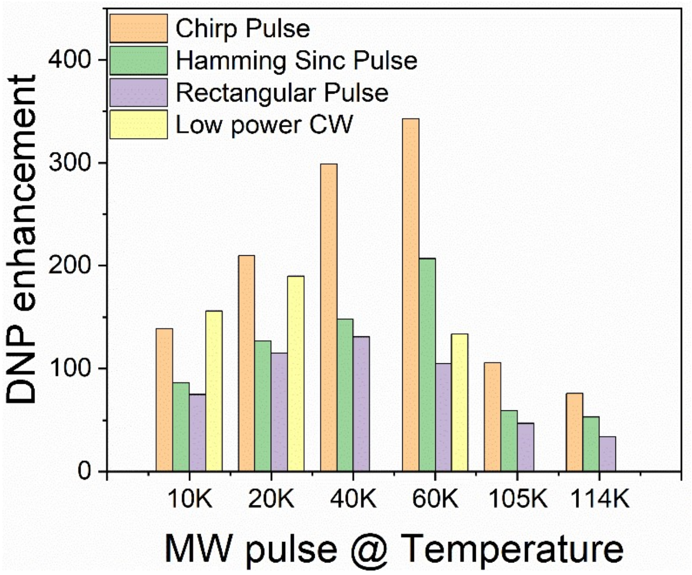

Supplementary Figure 1: Graph showing DNP enhancements achieved with cw radiation (200 mW) and different excitation pulses (B_1 ~ 15 G) using 50 mM 4-amino TEMPO as a polarising agent. In each case the parameters of the excitation were optimised to maximise the DNP enhancement. For each temperature the excitation bandwidth is comparable for three different types of pulses. Note that low power cw experiments were only performed at three temperatures (10K, 20K and 60K), and relatively high efficiencies were obtained at low temperatures.

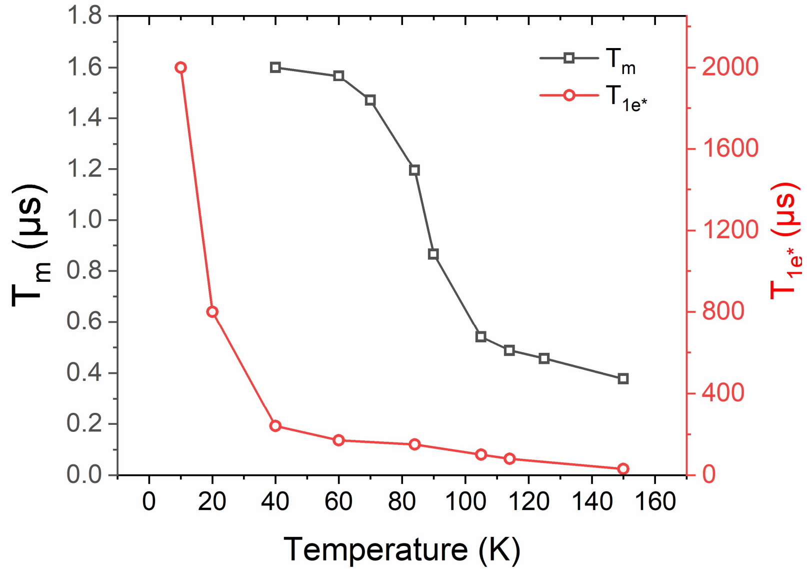

Supplementary Figure 2: Electron phase memory time T_m (black) and effective T_{1e*} (red) for narrowband pulses as a function of temperature for 4-amino TEMPO (50 mM) at 94 GHz. T_m was measured using Hahn echoes with soft pulses at the peak of the spectrum (black). Note the effective T_{1e*} is an effective electron

recovery time T_R, determined at different temperatures by fitting the spin echo height as a function of shot repetition time (SRT) using 1-exp(-SRT/T_R). All Hahn spin echo experiments were measurements with long pulses (100 ns – 200 ns) to reduce the effects of instantaneous diffusion. No significant change in relaxation rate were observed with further longer pulses. Note the specified T_{1e*} is an effective recovery time T_R and due to the combined effects of spectral diffusion and longitudinal relaxation.

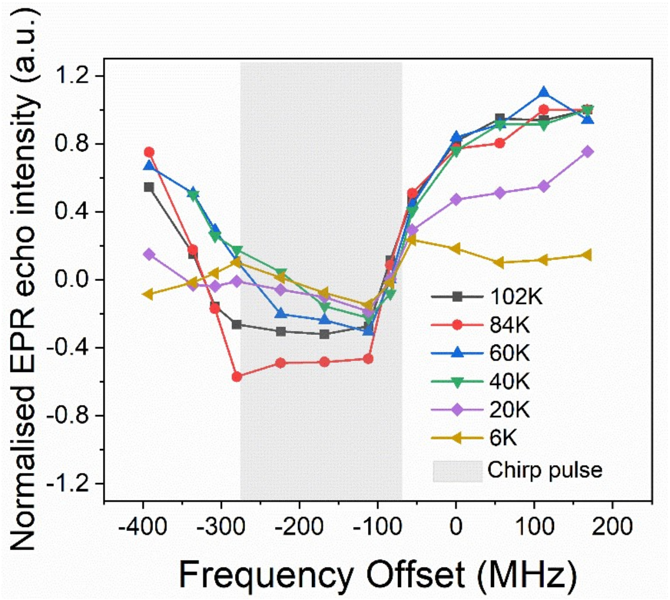

Supplementary Figure 3: Normalised ELDOR signal for 4-amino TEMPO where the spin echo was measured 4 μs after the DNP chirp pulse. The signal is normalised to the spin echo without chirp pulse at each frequency. The experiment was done using the repetition rate optimal for DNP enhancement. It should be noted that low repetition rates produced inversion efficiencies close to 90% with the chirp pulse and small increases in echo height were observed at 60K at 100 MHz offset.

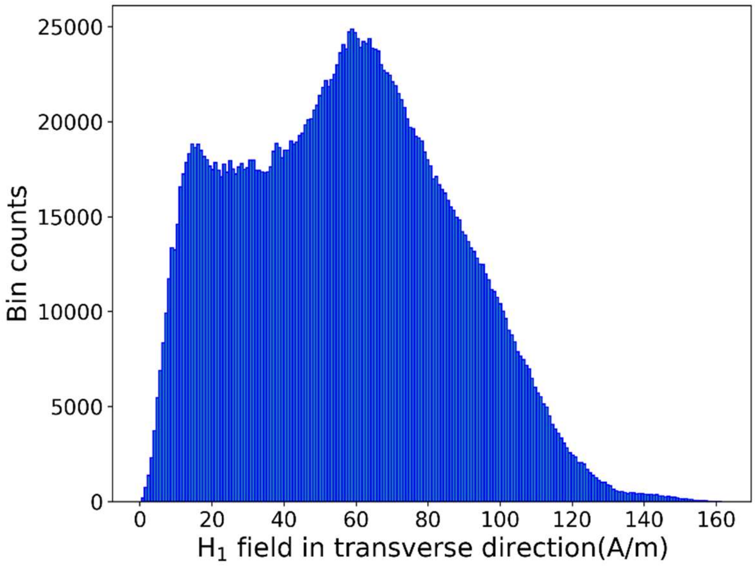

Supplementary Figure 4: Simulated transverse H_1 magnetic field intensity distribution across the sample volume assuming 1 W input power. Three million sampling points over the volume were chosen for the

histogram and the data was extracted from the electromagnetic simulation shown in Fig. 8 in the main text. In the pulse experiments described in this work the input microwave power at the sample is ~ 600 W and the experimental effective H_1 field is nearly 25 times larger than indicated in the figure.

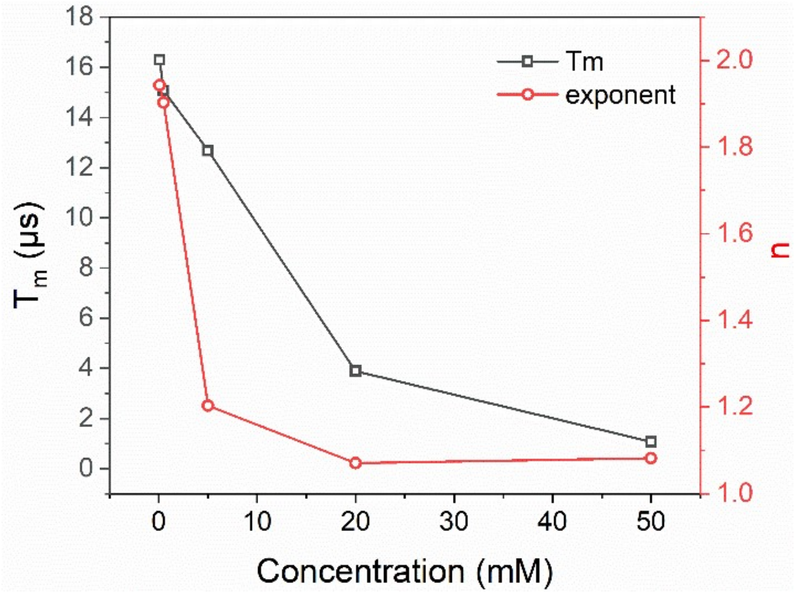

Supplementary Figure 5: Phase relaxation time T_m and exponent n as a function of TEMPO concentration, measured at Q band at 50 K using a Bruker ELEXYS E580 pulsed EPR spectrometer. T_m and n were calculated by fitting the experimental decay curve to the function $A\exp\left(-\left(x/T_m\right)^n\right)+C$. The data shows the reduction of T_m as concentration increases, and the change in underlying mechanism denoted by the change in exponent n. Soft pulses were used to reduce/eliminate the effects of instantaneous diffusion.

Supplementary Table 1: Average input microwave power required at each temperature for optimised DNP enhancement at W-band. Note that the microwave power at the sample is less than half of the value shown here due to the transmission loss.

Temperature (K)	10	20	40	60	85	100	115	150
Average power (W)	0.117	0.1911	0.546	0.6825	0.78	1.95	1.95	2.925

Supplementary Table 2: Relaxation properties of 50 mM 4-amino TEMPO in DNP juice at different temperatures at 94 GHz.

Temperature	10 K	20 K	40 K	60 K	84 K	105 K	114 K	150 K
T_m (μs)	--	--	1.60	1.56	1.00	0.54	0.48	0.37
T_{1e*} (μs)	2000	800	240	170	150	100	80	30
$T_{80\%SRT}$ (μs)	3333	1428	400	285	250	166	133	66
